# Illness Experiences in Young and Middle-Aged Patients With Dilated Cardiomyopathy on Chinese Social Media: Qualitative Study

**DOI:** 10.2196/76918

**Published:** 2026-05-05

**Authors:** Weijing Chen, Hanyu Xiao, Chenchen Feng, Jialin Li, Jinhua Jin, Ruimin Mu

**Affiliations:** 1 Nursing Department, Sir Run Run Shaw Hospital, Zhejiang University School of Medicine, 3 Qingchun East Road, Hangzhou, 310016, China, 86 13588056891

**Keywords:** dilated cardiomyopathy, young adults, qualitative research, social media, nursing

## Abstract

**Background:**

Dilated cardiomyopathy (DCM), characterized by ventricular dilation and systolic dysfunction, has a 10-year survival rate of 25%. The number of young and middle-aged patients with DCM is increasing, with progressive physical limitations and psychosocial distress substantially impairing quality of life. However, illness experiences in this population remain underexplored, particularly in non-Western contexts. Social media platforms such as Zhihu and Weibo offer a novel avenue for exploring patient narratives and peer support.

**Objective:**

A qualitative descriptive study was conducted to explore the multidimensional experiences of young and middle-aged Chinese patients with DCM through the social narratives shared on social media.

**Methods:**

A qualitative reflexive thematic analysis was conducted on 872 questions and responses from Zhihu, a Chinese Q&A platform. Data were extracted using a Python (Python Software Foundation) application programming interface, manually filtered, and analyzed with NVivo 14.0 (Lumivero). Thematic codes were developed iteratively, with researcher reflexivity and team discussions ensuring analytical rigor.

**Results:**

Five themes emerged: (1) bodily control loss (persistent sleep disturbances, severe respiratory compromise, and physical activity limitations), (2) enmeshed in emotional turmoil (anxiety over survival challenges, guilt over burdening family, and heterogeneous responses to death), (3) social and family role disruption (impacts on academic and career paths and family responsibility interruptions), (4) support needs in health care (urgent demand for information on diseases and treatments and desire for continuous care and guidance), and (5) life reconstruction—between disease and life (lifestyle modifications, exploring diversified treatment paths, peer support and experience sharing, and reconstructing the meaning of life).

**Conclusions:**

This study provides an interpretive account of the intertwined physical, psychological, and social experiences of young and middle-aged patients with DCM in China, emphasizing the influence of life-course stage and sociocultural context. Patients’ perceptions of family responsibilities, societal role expectations, and death shape their understanding of illness and their processes of meaning reconstruction. These findings underscore the importance of incorporating life-stage and culturally sensitive perspectives in future research and highlight the potential value of tailored psychological support and digital health platforms in improving access to illness-related information and ensuring continuity of care.

## Introduction

Dilated cardiomyopathy (DCM) is a diverse myocardial illness marked by ventricular dilation and reduced cardiac contractility [1,2]. Its pathophysiology is complex, involving genetic, viral, immunological, and metabolic factors. The World Health Organization (WHO) classifies DCM as a significant cardiovascular disorder that is a leading cause of heart failure, malignant arrhythmias, and sudden cardiac death [3,4]. Epidemiological studies show that patients with DCM have a 5-year survival rate of about 50%, which drops further after 10 years [5,6], with an estimated mortality rate of 5.9 per 100,000 people [7].

In addition to poor clinical results, patients with DCM commonly experience recurrent illness exacerbations or symptom deterioration, which are accompanied by significant psychosocial distress, such as sadness, anxiety, and low self-esteem [8]. However, psychosocial assistance within current care pathways is noticeably insufficient. According to the UK Cardiomyopathy Charity’s Cardiomyopathy Care Report (2023), more than half of adult respondents reported difficulties with emotional regulation in the previous 6 months as a result of cardiomyopathy, while only 9% received any form of psychological support during their care [9].

Notably, as heart failure develops at a younger age, so does the affected population, with the prevalence among young and middle-aged individuals increasing by around 50% [10,11]. Young and middle-aged patients with DCM have significant clinical and psychological features. Disease progression and outcomes may differ from those seen in older individuals [12], and this demographic faces more complex work, familial, and societal demands when dealing with chronic illness. Adults with DCM report significantly lower quality of life in the physical, emotional, and social domains, according to a cross-sectional study by Steptoe et al [13], highlighting the need for specialized psychological interventions and encouraging counseling [14]. However, the majority of current research has concentrated on improving clinical treatment approaches and clarifying pathophysiological mechanisms [15,16]. The development of patient-centered and precision care models is hampered by the lack of research on the illness experiences and psychosocial needs of young and middle-aged patients with DCM, despite the fact that psychosocial adaptation has been studied in younger and middle-aged populations with conditions like diabetes and breast cancer [17,18]. A thorough understanding of patient experiences may be hindered by the limitations of traditional data-gathering techniques like interviews, which frequently face issues such as recollection bias and contextual constraints [19].

The rapid development of digital health technologies presents fresh opportunities to close this disparity. In today’s society, the internet has emerged as a crucial medium for health communication [20]. These platforms enable patients to interact with others who are dealing with the same medical issues, offering guidance and emotional support that would not be accessible in conventional health care settings. Additionally, they provide a way for patients to express their individual health journeys outside the limitations of formal clinical settings. Nearly 69.2% of China’s 1.108 billion internet users as of December 2024 were young and middle-aged people [21]. This group is more likely to use social media sites like Weibo and Zhihu to seek peer support and discuss health-related experiences. About 15% of the content on Zhihu, one of the biggest Q&A sites in China [22], relates to discussions among its 14.2 million subscribers [23,24] and over 100 million monthly active users. Researchers can record a variety of real-time narratives of lived experiences that might not surface in conventional clinical interviews thanks to this realistic, peer-to-peer contact, which offers insightful information about the multifaceted experiences of young and middle-aged persons with DCM.

The concept of “illness experience” is critical for understanding the psychosocial and emotional effects of DCM on patients. The theoretical underpinning for this notion comes from the Engel biopsychosocial model of illness [25], which holds that illness is more than just a biological condition; it is a complex interaction of biological, psychological, and social elements that affect patients’ total well-being. This paradigm highlights the necessity of taking into account not only medical symptoms but also how people adapt emotionally and socially to their condition. In this study, illness experience is defined as the multidimensional and dynamic process by which patients perceive, understand, and respond to the physical, emotional, and social demands of living with a chronic illness. This encompasses not only obvious physiological symptoms but also emotional suffering, shifts in social roles, and coping techniques used in reaction to the disease. By using this approach, we hope to gain a better understanding of how patients with DCM deal with the physical, emotional, and social aspects of their illness.

As a result, this study examines conversational posts written by young and middle-aged patients with DCM on the Zhihu platform to learn about their illness experiences. The study uses qualitative research methodologies to explore the physiological, psychological, and social experiences of young and middle-aged patients with DCM through their online narratives.

These findings may serve as a theoretical foundation for the development of targeted therapies and support programs aimed at improving self-management and quality of life in young and middle-aged patients with DCM.

## Methods

### Research Design

This descriptive qualitative study used media-based naturalistic narratives to capture the illness experiences of patients with DCM, preserving the richness and depth of their perspectives. This approach offers valuable insights into the development of targeted interventions for patients with DCM. The study adhered to the COREQ (Consolidated Criteria for Reporting Qualitative Research) guidelines (Checklist 1) [26].

### Data Collection

On March 30, 2025, we used Python (Python Software Foundation) to collect data on DCM among young and middle-aged adults on Zhihu via the platform’s application programming interface. The search included phrase combinations associated with DCM and the target age group, such as “young people + dilated cardiomyopathy,” “middle-aged dilated cardiomyopathy,” “youth dilated cardiomyopathy,” and “young and middle-aged adults dilated cardiomyopathy.” To balance data richness with analytical practicality and account for application programming interface limits, we collected only the top 50 threads per search phrase, as determined by Zhihu’s default popularity algorithm. To reduce data loss, the number of collected replies for each thread was manually validated against the platform’s response count. This procedure yielded 200 question threads with 8727 user responses.

To create a high-quality collection of patient experience narratives, a manual screening process was performed on the initially collected 200 questions and 8727 associated replies using predetermined inclusion and exclusion criteria. Two researchers screened independently, and conflicts were settled by a third reviewer through consensus or arbitration. The inclusion criteria were as follows: (1) confirmation of patient identity, defined as explicit self-reporting of a DCM diagnosis (eg, “I was diagnosed with dilated cardiomyopathy”) [27]—responses describing relatives with DCM were included only if they evolved into detailed first-person accounts of the respondent’s own illness experiences; (2) age eligibility, with explicit indication that the respondent was aged 18 to 59 years, with age determined from self-reported information in the posts; (3) content relevance, in which responses were first-person narratives describing personal experiences with DCM, including symptoms, psychological states, and disease-related life impacts; and (4) temporal relevance, defined as responses posted between April 2020 and March 2025, corresponding to the 5 years preceding data collection.

Exclusion criteria included (1) insufficient narrative depth, defined as responses containing fewer than 100 words or content too vague to support thematic analysis; (2) nonexperiential or informational content, such as popular science explanations, reposted medical information, comments from health care professionals, caregivers, or other third parties, as well as responses limited to motivational or general well-wishing content; and (3) commercial or promotional material, including product recommendations, institutional advertisements, embedded promotional links, or text exhibiting explicit marketing intent, as identified by assessing writing purpose, content balance, and the respondent’s posting history. Throughout the analytic process, the research team engaged in iterative conversations and reflexive deliberation to refine codes, challenge interpretations, and improve analytic rigor.

Zhihu enables multilingual content, but the platform is largely in Chinese. All search queries were in Chinese, and the screening process was based on Chinese-language proficiency; therefore, all included data consisted of Chinese-language texts. After deleting irrelevant and duplicate questions and responses, the final dataset consisted of 872 postings with valid patient narratives that met all inclusion criteria. This multistage screening method ensured a true representation of the intended patient group while reducing any confounding factors and bias.

### Analysis Strategy

Data were examined using reflexive thematic analysis, a qualitative method for identifying shared patterns of meaning throughout a dataset. This method was chosen for its conceptual flexibility and emphasis on researchers’ active participation and reflexive posture throughout the coding and theme generation phases [28]. This approach views researchers as active contributors to knowledge development rather than passive spectators. The analysis followed Braun and Clarke’s [29] 6-phase framework, which included data familiarization, initial coding, topic generation, theme review, theme definition and naming, and report authoring. To retain semantic nuances, cultural connotations, and contextual coherence, all analyses were carried out in Chinese, the primary language spoken by both the researchers and the study participants. Following the completion of the analysis, illustrative quotations and synthesized findings were translated into English by the first author for reporting purposes. NVivo (version 14.0; Lumivero) was used to help with data management, coding, and theme creation. Throughout the analytic process, the research team engaged in iterative conversations and reflexive deliberation to refine codes, challenge interpretations, and improve analytic rigor.

### Analysis Procedure

Researcher WC led and directed all analytic phases, with other members of the research team providing input on crucial analytic conclusions via a series of crystallization talks. This collaborative process facilitated multidisciplinary reflection while also increasing the depth and rigor of the study. In the first phase (data familiarization), WC manually cleaned the information, deleting all private usernames and references to specific geographic regions to protect user privacy. Throughout this procedure, the full dataset was reviewed multiple times to achieve immersion, and analytic reflections were recorded in a reflexive log (Multimedia Appendix 1). The second phase (initial coding) involved 2 researchers independently coding 20% of a randomly selected portion of the data. The coding results were then addressed at the first crystallization conference, where the research team presented preliminary interpretations and perspectives on potential meanings in the data. Building on these conversations, WC used descriptive coding on the remaining data. This method generated 176 initial codes. A second crystallization meeting was held to review these preliminary codes. Following the meeting, WC merged overlapping codes, deleted redundant or conceptually shallow codes, and modified the code set to increase analytic focus. This continual refinement resulted in the final set of 52 codes (Multimedia Appendix 1). In the third phase (theme generation), WC organized the refined codes into larger conceptual patterns and created 5 tentative themes. During the fourth and fifth phases (theme review and definition), the research team held a third crystallization meeting to assess the themes’ coherence with respect to the overall dataset and to refine theme boundaries. The themes were examined, improved, and given final names that best matched the data’s main narratives (Multimedia Appendix 1).

### Reflexivity

Throughout the study, the research team engaged in ongoing reflexive practice to investigate how personal beliefs, professional backgrounds, and underlying assumptions influenced the research process and the interpretation of findings. The team included personnel with a variety of clinical and academic positions, including a clinician specialized in chronic disease management (YP), a head nurse in cardiology (JJ), a cardiology education nurse (CF), and master’s-level nursing graduate students. JJ has considerable clinical experience caring for patients with cardiovascular problems, whereas CF specializes in patient communication and cardiology-focused health education, allowing him to have a thorough understanding of patients’ views and needs. The principal researcher’s overall study program focuses on understanding the lived experiences of people with cardiovascular diseases, with a special emphasis on the bidirectional linkages between psychological and physiological processes. In addition, WC and HX received professional training in reflexive thematic analysis and qualitative data management using NVivo software. These diverse professional responsibilities and experiential backgrounds were identified as possible influencers on data interpretation. At the same time, they were considered a source of analytic strength, providing complementary views and boosting both the depth and credibility of the study findings [30].

### Rigor

This study followed best-practice guidelines proposed by Korstjens and Moser [31] to ensure trustworthiness in qualitative research, with particular attention to the principles of transferability, credibility, and reflexivity. To enhance credibility, a collaborative analytic approach involving multiple researchers was adopted. During the coding process, 2 researchers independently coded approximately 20% of the dataset and subsequently discussed discrepancies until consensus was reached, thereby reducing potential bias arising from a single researcher’s interpretation. In addition, regular team meetings were conducted throughout the analytic process to continuously review and refine the coding framework and the development of themes. To improve transparency and traceability of the analytic process, examples of corresponding codes and their thematic categorization are provided in Multimedia Appendix 1. Furthermore, reflexive journals were maintained throughout the study to document researchers’ positions and consider how these perspectives might influence data interpretation, thereby further strengthening reflexivity and the methodological rigor of the study.

### Ethical Considerations

Research using social media data mining is in a gray area, necessitating not only institutional ethical control but also careful consideration of participant privacy, data use, and the vulnerability of individuals sharing personal health experiences online. Although Zhihu users agree to the platform’s privacy policy [32] and understand that their content may be made public, those who upload illness narratives may not foresee their use in academic research. As a result, the gathering and analysis of publicly available data in this study were found to be ethically acceptable, but obtaining individual informed consent was deemed impractical. All data were obtained solely from Zhihu, with no access to nonpublic or restricted content, and all procedures adhered to the platform’s terms of service and data use policy. This study was reviewed by the ethics committee of Sir Run Run Shaw Hospital, Zhejiang University School of Medicine, and was determined to be exempt from formal ethical approval, with a waiver of informed consent granted due to the use of publicly available, nonidentifiable data. Several protections were put in place to reduce the risk of harm. To decrease the possibility of indirect identification, usernames were replaced with unique identification codes, and textual quotes were paraphrased or deidentified [33,34]. Raw data were only accessible to the research team and were kept on an encrypted server. These parameters struck a balance between the scientific usefulness of evaluating publicly published illness narratives and respect for participants’ privacy, dignity, and vulnerability.

## Results

### Data Description

A total of 872 questions and responses relevant to young and middle-aged patients with DCM were analyzed. Five themes emerged from the analysis: (1) loss of bodily control, (2) enmeshed in emotional turmoil, (3) social and family role disruption, (4) support needs in health care, and (5) life reconstruction—between disease and life. Textbox 1 lists the 5 core themes and their subthemes. Table 1 lists the number of questions and responses for each topic.

Textbox 1. **The 5 core themes and subthemes.**

**Theme 1: bodily control loss**

- Persistent sleep disturbances
- Severe respiratory compromise
- Physical activity limitations

**Theme 2: enmeshed in emotional turmoil**

- Anxiety over survival challenges
- Guilt over burdening family
- Heterogeneous responses to death

**Theme 3: social and family role disruption**

- Impacts on academic and career paths
- Family responsibility interruptions

**Theme 4: support needs in health care**

- Urgent demand for information on diseases and treatments
- Desire for continuous care and guidance

**Theme 5: Life reconstruction—between disease and life**

- Lifestyle modifications
- Exploring diversified treatment paths
- Peer support and experience sharing
- Reconstructing the meaning of life

**Table 1.** Details of the data, including the number of questions and responses for each theme.

Themes	Questions and responses (N=872), n (%)
Bodily control loss	96 (11)
Enmeshed in emotional turmoil	164 (18.8)
Social and family role disruption	110 (12.6)
Support needs in health care	279 (31.9)
Life reconstruction—between disease and life	223 (25.7)

### Thematic Analysis

We identified 5 core themes and their subthemes from the data, which highlight the multidimensional challenges and coping strategies experienced by young and middle-aged patients with DCM. These themes encompass physical impairments, psychological distress, social role disruptions, health care support needs, and corresponding adaptive strategies, including life-rebuilding coping behaviors (Textbox 1).

#### Theme 1: Bodily Control Loss

Patients often describe a rapid decline in physical function due to the disease, marked by irreversible damage to physiological functions and significant limitations in daily activities.

##### Persistent Sleep Disturbances

Most respondents reported being unable to lie flat due to heart failure symptoms, forcing them to adopt a seated position for sleep, which significantly impaired sleep quality. Some patients rely on sedative medications to achieve brief periods of sleep, while others compare their current physical state to their predisease condition, leading to a strong sense of loss of control:

*I just can’t fall asleep—my heart feels like it’s about to burst. It’s not like it’s skipping beats; it feels more like I’m being smothered. That bubbling, squeezing heartbeat sound—it’s terrifying.*

[P45]

*I can’t get proper rest, but if I take Stilnox, it helps me squeeze in maybe 3 hours of sleep.*

[P112]

*What kills me is remembering how it used to be. I used to sleep straight through the night—now I wake up gasping, scrambling to sit upright just to breathe.*

[P83]

##### Severe Respiratory Compromise

Patients with DCM may have a low cardiac ejection fraction and reduced blood pumping ability, potentially leading to pulmonary congestion and edema, which can cause dyspnea. Severe respiratory deprivation may also trigger a sensation of suffocation, akin to drowning:

*When my heart acts up, it feels like death knocking. My chest tightens, pounds, and aches.*

[P27]

*Lying flat turns me into a wheezing mess—I gasp like I’m underwater, choking on foamy phlegm.*

[P156]

##### Physical Activity Limitations

Respondents generally reported a gradual decline in their ability to perform daily activities due to DCM symptoms. Basic tasks—such as dressing, climbing stairs, and walking—required considerable effort. These functional impairments led some patients to reduce their activity levels, with some even considering staying home as their only option:

*It takes me 3 separate attempts just to take off a shirt now—every little thing feels like a battle against my own body.*

[P09]

*It feels like I’ve been fast-forwarded through time. Climbing 2 flights of stairs leaves me breathless, like I just ran a marathon. A 1-kilometer run that I once completed easily now feels impossible, and even stairs have become an insurmountable obstacle.*

[P191]

*I sweat a lot while sleeping, and I feel very weak. I’ve been lying in bed at home, afraid to go outside even for a walk.*

[P63]

### Theme 2: Enmeshed in Emotional Turmoil

The study revealed the multifaceted psychological and emotional experiences that patients face as they adapt to the disease, as outlined in the following subsections. A total of 3 subthemes were identified.

#### Anxiety Over Survival Challenges

Some patients experience anxiety and helplessness when confronted with the pressures of survival, with financial strain and career limitations being major sources of distress. On one hand, the high cost of treatment contrasts with the family’s insufficient financial resources to support the expensive treatment. On the other hand, patients face employment difficulties due to physical decline, which prevents them from securing a stable income:

*We just can’t keep up with the treatment costs—it’s draining money we don’t have. How long do I even have left? A year? Two? Honestly, I’m terrified I could be gone tomorrow.*

[P134]

*I finally started feeling a bit stronger and tried to imagine rebuilding my life. But how? You can’t rely on your parents forever. Then it hit me—society can be ruthless. Suddenly, I lost all hope.*

[P72]

#### Guilt Over Burdening Family

Patients with DCM often depend on their family members for care and companionship. However, some middle-aged patients, despite being in their prime years, rely on older adult parents for support, leading to feelings of guilt and self-blame. Some patients even choose to end intimate relationships to avoid burdening their partners, while others express guilt about the cost of treatment. Additionally, concerns regarding genetic risks associated with childbearing were also mentioned:

*What I feel most sorry about is my parents—I can no longer support them in their old age.*

[P38]

*After I got sick, I didn’t want to burden my boyfriend, so I ended the relationship that had lasted from graduate school through the start of my career.*

[P155]

*Most of my family’s income goes toward my treatment. I feel deeply sorry for them.*

[P102]

*I know that heart disease may be passed on to the next generation. If this disease is hereditary, I won’t have children. Even if I did, I wouldn’t be able to take care of them.*

[P89]

#### Heterogeneous Responses to Death

Confronted with a limited life expectancy, patients demonstrate individualized coping strategies and emotional responses. Most respondents reported difficulty accepting the prospect of living only 5 more years, describing feelings of fear and despair while living with what they referred to as the “5-year curse”:

*I never thought I’d get such a severe illness. It’s really hard to accept… What should I do now?*

[P167]

*I once had a Holter monitor test, and my heart rate dropped to 34 beats per minute while I was sleeping. It felt like I might not wake up. I’m only 20 years old, not yet married, and I still have hope for life. I don’t want to die yet.*

[P13]

Some patients calmly accept the reality of facing death while maintaining an optimistic attitude:

*I don’t know if I can live for another 10 years. I plan to follow the doctor’s advice, take my medication on time, and live happily each day.*

[P94]

*I’ve heard stem cell technology is going to be applied clinically soon. I’ll keep fighting to live. Maybe one day medical technology will make a breakthrough!*

[P182]

Some patients engage in avoidance behaviors, attempting to challenge the medical diagnosis or refusing medical examinations to escape the prospect of death:

*After my last scan showed an ejection fraction of 23%—a tiny bump from last time—I quit my meds. Why bother with checkups? Ignorance feels safer than bad news.*

[P41]

*Is there a high misdiagnosis rate for DCM? Is there another possibility?*

[P109]

### Theme 3: Social and Family Role Disruption

Following the onset of disease, younger patients frequently experience disruptions to their educational and early career trajectories, whereas middle-aged patients are more likely to encounter strain or disruption in family roles due to the substantial responsibilities associated with being primary household providers.

#### Impacts on Academic and Career Paths

The academic progress of some young patients was significantly hindered by their illness. Some patients who were pursuing a master’s degree found that their health condition inevitably became a major factor in their future prospects:

*Because of this disease, I had to take a year off from school, and since then, my grades have dropped dramatically.*

[P55]

*I’m a graduate student, in the prime of my life, but being diagnosed with DCM is a huge catastrophe.*

[P112]

Additionally, the impact of their health condition often forces difficult decisions, such as changing jobs or facing unemployment:

*Regardless of the risk of the operation, it is difficult to successfully match a suitable heart donor. So I chose conservative treatment and stayed at home without looking for a job.*

[P29]

*I used to love cosmetics, and before the disease, I was a cosmetic formula designer for a big company, but after the illness, I was let go.*

[P173]

#### Family Responsibility Interruptions

Many middle-aged patients face the dual responsibilities of supporting both their older adult parents and raising young children. However, due to their health conditions, they are often unable to fulfill the obligations associated with these roles, leading to feelings of powerlessness:

*My children are still young, and my parents need support. How can I just give up?*

[P87]

*The more often I go to the hospital, the more afraid I become. I’m young, but I have both elderly parents and young children. I feel overwhelmed, but I have no choice but to face it.*

[P142]

### Theme 4: Support Needs in Health Care

Illness experiences shared by young and middle-aged patients on the Zhihu platform highlight challenges in navigating health care and obtaining disease-related information. Some patients reported delayed medical consultations attributable to limited disease-related knowledge, whereas others expressed heightened concerns regarding disease information and described difficulties in accessing continuous care and professional guidance.

#### Urgent Demand for Information on Diseases and Treatments

During the early stages of symptom onset, young and middle-aged patients with DCM often underestimate the seriousness of their condition due to limited disease-related knowledge. After receiving a diagnosis, many actively seek information on Zhihu regarding disease etiology, symptoms, dietary recommendations, and treatment options, with the aim of gaining a clearer understanding of disease progression and management strategies:

*At 34, my initial symptoms felt like a cold, and I had a cough for 3 months. I thought it was just a flare-up of my chronic pharyngitis. But when I started coughing up pink sputum and couldn’t lie down to sleep, I realized something was wrong and went to the hospital.*

[P121]

*I’m 28 years old and have been suffering from this disease for 5 years. I hope for a miracle—does anyone know about the latest treatment progress?*

[P154]

*I am 49 years old and was diagnosed with DCM last week. What should I do now? Should special dietary restrictions be noted?*

[P88]

#### Desire for Continuous Care and Guidance

Some patients face a lack of continuous care during the process of seeking medical treatment:

*The local hospital doesn’t have doctors who can treat DCM, so we had to rely on larger hospitals.*

[P192]

*I am 25 years old and have DCM with stage IV heart failure. At my worst, my ejection fraction was 9%, and I had fluid accumulation in multiple parts of my body. I visited many hospitals, but they all gave me a poor prognosis and advised transferring to another hospital.*

[P33]

### Theme 5: Life Reconstruction—Between Disease and Life

Amid the shadow of medical prognoses, young and middle-aged patients with DCM actively reconstruct their lives through multidimensional adaptive strategies. These patients adopt various approaches—ranging from strict self-discipline in diet and lifestyle to exploring integrated treatments that combine Western and traditional Chinese medicine—seeking to balance disease management with daily living. Additionally, through mutual support with fellow patients, they share experiences, foster hope, and engage in psychological struggles that reshape their understanding of life’s meaning.

#### Lifestyle Modifications

Confronted with the threat of DCM, patients adjust their daily routines to manage the physical changes caused by the disease. Many quit smoking and drinking, maintain a regular schedule, follow a lighter diet, take medications on time, and engage in moderate exercise:

*I’ve quit smoking and cut out alcohol completely—no more late nights either. In bed by 10, up at 7 sharp, meds on schedule. Even added a nightly 30-minute walk after dinner.*

[P117]

*Since my diagnosis, I’ve turned into a health-conscious person—early bedtimes, bland meals, everything. It feels like I’m living like a retiree at 40.*

[P62]

#### Exploring Diversified Treatment Paths

In recent years, integrated treatment approaches that combine Western and traditional Chinese medicine have offered new hope to patients. Young and middle-aged patients with DCM are open to exploring various therapeutic options, hoping to find an effective cure through diverse treatment pathways:

*I’m currently taking Western medicine with supplementary traditional Chinese medicine treatment.*

[P179]

*A local traditional Chinese medicine doctor recommended making tea from red dates and lotus seeds, although I don’t know if it’s effective, I thought it couldn’t hurt to try.*

[P25]

*After I decided to adopt an integrated traditional Chinese and Western medicine treatment, both mitral and tricuspid regurgitation improved significantly.*

[P2]

#### Peer Support and Experience Sharing

Throughout their journey with DCM, patients actively engage with others on platforms like Zhihu, offering life advice, treatment tips, and psychological support. They share real-life experiences to inspire hope in others. Peer support and the exchange of experiences have become vital sources of strength in combating the illness. Emotional support from fellow patients, particularly those of a similar age, is especially significant.

*If you have heart problems, it’s important to eat light meals and stick to a regular routine. Also, make sure to stay warm. I feel discomfort in my heart when the seasons change or when I am in cold wind for too long.*

[P141]

*This disease may seem scary, but we need to manage it wisely. Take it seriously by following your doctor’s treatment plan while staying positive. Remember, this disease does not always require a heart transplant.*

[P166]

*I was diagnosed with this disease when I was 18. Don’t give up on treatment, especially if you’re around my age. We still have a chance for recovery. Let’s live our lives to the fullest!*

[P104]

#### Reconstructing the Meaning of Life

Under the looming shadow of death, many patients reassess the meaning of life. Some attempt to accept their mortality by engaging in meaningful activities during their remaining time. Others, after confronting the brink of life and death, find new meaning in their lives by focusing on family and love, which leads to posttraumatic growth. Notably, some patients shift the focus of their lives from self-actualization to familial responsibility, forming a new ethical contract of “living for others”:

*I don’t want to die like this; I want to fight. I want to do meaningful things in the time I have left, to love those who care about me and make the most of every minute.*

[P93]

*After experiencing a near-death experience, I want to cherish my life even more and treat my parents with greater respect.*

[P132]

*Living well isn’t just about me anymore. I now feel like my life belongs to my children, my younger sister, and my parents. I cannot give up easily because they are always there for me.*

[P07]

## Discussion

### Principal Findings

This study presents the first systematic analysis of illness experiences among young and middle-aged patients with DCM. Using reflexive thematic analysis, 5 overarching themes with associated subthemes were identified through the analysis of patient narratives. Themes 1 to 3 capture the complex aspects of patients’ illness experiences, stressing issues in the physiological, psychological, and social realms. The physical restrictions mentioned in theme 1 frequently increase the emotional distress presented in theme 2, and changes in social roles, as discussed in theme 3, exacerbate both the physical and psychological burdens. Theme 4 describes patients’ challenges in obtaining disease-related information and accessing ongoing treatment. Finally, theme 5 focuses on the active processes by which patients translate their lived experiences and disease-related challenges (as themes 1‐4) into actual activities and the creation of personal meaning.

The subthemes of theme 1 (bodily control loss) are interconnected. Severe respiratory compromise causes recurrent sleep disturbances because breathing difficulties prevent patients from sleeping flat, thereby reducing sleep quality. Sleep deprivation, in turn, causes weariness and exacerbates respiratory problems, further restricting physical activity. The interplay of these subthemes emphasizes the compounding impact of disease symptoms on patients’ daily lives, as well as the physical hardships they bear. Among these multidimensional experiences, abrupt bodily changes are frequently the first and most noticeable effects felt by young and middle-aged individuals. Compared to older patients, these individuals experience a “sense of bodily betrayal”—a rapid deterioration in heart function that causes deep loss of body control and disrupts life goals. This behavior has rarely been described in previous DCM investigations, likely because younger patients compare themselves to healthier peers, exacerbating perceived loss and causing “asynchronous” life-course stress that impedes role adaptability. Charmaz [35] also found physical alienation among adults with chronic disease, accompanied by submission to illness. Comparable experiences occur in adolescent and young adult patients with cancer, who feel alienated due to treatment-related physical changes shaped by social contexts [36]. Body image disturbances in DCM reflect both functional decline and broader psychosocial consequences. Health care providers should promote body acceptance in clinical or community settings by conducting body image–focused psychological screenings and offering targeted interventions. Cognitive behavioral therapy has been shown to improve self-acceptance and alleviate distress in people with chronic illnesses [37].

When the body is no longer reliable, its impact often extends to the patient’s existing social roles and responsibilities, leading to deeper psychological distress. The twin demands of high treatment expenses and employment obstacles frequently intensify anxiety, as evidenced by Abidan’s case study of young and middle-aged patients with DCM [38]. Patients frequently experience anxiety as a result of their failure to fulfill tasks as economic providers or caregivers, which causes feelings of guilt for “burdening” their family members. This ethical quandary has also been observed in investigations of patients receiving maintenance hemodialysis and survivors of stroke in China [39,40]. Patients’ feelings of shame are ingrained in the familial and societal institutions they inhabit. While the family plays a significant role in chronic disease care across cultures [41], its specific expressions and moral consequences differ. In the Chinese setting, filial piety is fundamentally normative, requiring adult children to provide financial, emotional, and end-of-life care for their parents, and failing to do so may be viewed as “unfilial” [42,43]. Rigid expectations about the “breadwinner” role exacerbate shame in young and middle-aged adults. In this study, some patients even ended personal connections to alleviate the perceived burden on their families, demonstrating “ethically driven guilt” aimed at collective family well-being [44]. These findings also emphasize how the cultural environment influences the emotional experiences of patients with chronic disease [45]. Accordingly, nursing interventions should respect cultural differences, take into account family relational networks, and give tailored, culturally sensitive assistance. Family gatherings, intergenerational communication, and family life planning might help patients renegotiate role expectations and reduce guilt and survival anxiety.

Under prolonged stress, patients inevitably confront the issue of death. Patients who are stressed for an extended period of time are inevitably confronted with the possibility of death. The majority express acute terror, while a minority remain optimistic, hoping for medical breakthroughs. Threatening medical statistics, such as “5-year survival rates,” are sometimes viewed as a “countdown to life,” which heightens anxiety. In traditional cultural contexts, some patients adopt a fatalistic attitude, which may impact treatment adherence and psychological adjustment [46]. A few people use negative coping mechanisms, like denial or avoidance. These disparate views, driven by fear and avoidance, reflect both a lack of scientific understanding and an insufficient focus in clinical practice on the death-related concerns of young and middle-aged patients with DCM [47]. Without adequate emotional support and opportunities for discussion, negative cognitions can be reinforced by recurrent symptom experiences, resulting in a “poor prognosis—fear of death” loop. Therefore, incorporating assessments of patients’ views on death into routine care is critical for understanding patients’ perspectives and correcting cognitive biases. Developing multimedia-based death education for young and middle-aged patients with DCM may improve their psychological well-being [48].

It is vital to highlight that patients’ illness experiences vary greatly depending on their life stage. While a group examination of young and middle-aged patients with DCM can indicate common characteristics, social roles and psychosocial obstacles vary by developmental stage. Middle-aged patients frequently have family, economic, and caregiving duties, with disease-related disruptions to job and financial stability causing shame and social role crises. Young patients, on the other hand, face abrupt pauses in their education, careers, or personal relationships, as well as physical limitations that impair their self-esteem and future expectations. Tutelman et al [49] observed that adolescents and young adults are at a key developmental stage, and life-threatening illnesses cause distinct psychological demands that necessitate tailored interventions. Young and middle-aged patients with DCM, unlike adolescent and young adult patients with cancer, do not receive adequate psychological assistance for relationship and social role reconstruction, putting them at risk of social isolation and developmental suffering [50,51]. Evidence linking social integration in adults to all-cause and cardiovascular mortality [52] emphasizes the significance of taking life stage into account when treating psychosocial needs. Differentiated support can help people retain social participation and role continuity, which improves their quality of life. This study classified patients generally into young and middle-aged groups; further age stratification may reveal significant differences.

Furthermore, throughout medical therapy, young and middle-aged patients with DCM frequently report difficulties in obtaining adequate disease information and continuity of care, indicating a lack of disease awareness and difficulties in coping with the unpredictability of a sudden chronic condition. The diagnosis disrupts established life plans, increasing susceptibility to symptom variations and treatment outcomes. Uncertainty about disease progression and prognosis contributes to increased anxiety and a sense of loss of control. Our findings show that patients actively seek disease-related information to better understand their condition and restore a sense of control, which is especially important after discharge due to long-term self-monitoring and limited access to professional help. Digital health solutions have developed as instruments for addressing health care system issues and increasing universal health coverage [53]. Future interventions should assure ongoing delivery of intelligible, evidence-based information via social media content, supplemented by remote consultations or regular follow-ups, to assist patients in developing a stable understanding of their disease while reducing fear and feelings of powerlessness.

Despite numerous hurdles, DCM does not disrupt life’s order but rather encourages the construction of a new one [54]. Patients rebuild their lives by changing their behaviors, researching treatment choices, and seeking peer support, thereby redefining their sense of purpose and exhibiting resilience. Young and middle-aged patients frequently reduce anxiety by sharing their experiences on the Zhihu platform, likely due to the limited options for ongoing communication and emotional support in official health care settings. Peer support therapies have been proven to improve heart function markers in middle-aged patients with heart failure [55], implying that online peer groups may offer both emotional support and practical guidance. Familial duties also influence life reconstruction, with many patients changing their focus away from personal development and toward helping and safeguarding family members. This reflects the family-centered value orientation in Chinese culture [56], contrasting with the individualistic goals common in Western societies [57]. These findings highlight the need for culturally sensitive psychological and nursing interventions that help patients balance disease management with life responsibilities.

### Strengths and Limitations

This is the first qualitative study to examine the illness experiences of young and middle-aged patients with DCM, filling a knowledge gap and providing insights into their multidimensional experiences spanning physiological, psychological, and role-adjustment dimensions. Our findings expand the biomedical focus of DCM research, offering health care providers a deeper understanding of patient needs and enhancing the effectiveness of nursing care. Unlike previous studies, this research highlights patients’ developmental life stages and cultural context, addressing the lack of psychosocial research in non-Western populations.

Nonetheless, the study contains drawbacks. Data were gathered from a single social media network, which may have biased the results toward technologically literate and emotionally involved consumers. In addition, patients were classified generically as “young” or “middle-aged,” with no tighter age stratification, thus limiting the examination of within-group variations. Furthermore, in this study, fewer individuals reported the duration of their disease. Instead, their accounts focused primarily on their experiences during the illness. As a result, the absence of accurate information about the length of the illness may hinder a thorough understanding of how disease progression affects patients’ lives.

### Recommendations for Further Research

Future research could expand in several directions. First, similar qualitative studies across diverse health care settings and cultural contexts are needed to deepen the understanding of the illness experiences and challenges faced by young and middle-aged patients. Second, more detailed age stratification or developmental stage frameworks should be adopted to collect more comprehensive data on factors such as the duration of the disease, in order to explore the similarities and differences in psychological and social challenges, role conflicts, and the construction of life meaning between young and middle-aged patients. Third, longitudinal qualitative designs could capture dynamic changes in patients’ cognition, emotions, and coping strategies throughout disease progression. Finally, given the prominence of death in patients’ narratives in this study, future research could focus on young and middle-aged patients’ understanding of death, providing an empirical basis for refining theoretical frameworks.

### Implications and Conclusion

This study provides a qualitative analysis of the multidimensional illness experiences of young and middle-aged patients with DCM in China. Findings indicate that physical decline, psychological distress, and social role crises are closely intertwined, shaped by patients’ life stages and cultural context. Family responsibilities, societal role expectations, and perceptions of death collectively influence patients’ understanding of their illness and the reconstruction of life meaning. Theoretically, the study highlights the importance of examining the experiences of young and middle-aged patients with DCM within a biopsychosocial-cultural framework, offering a foundation for future research, supportive interventions, and cross-cultural chronic disease management. Practically, clinical interventions should account for patients’ developmental stages and cultural contexts, provide tailored psychological support, and leverage digital health platforms to enhance comprehension of disease information and ensure continuity of care.

## Supplementary material

10.2196/76918 Multimedia Appendix 1 Analysis procedure record.

10.2196/76918 Checklist 1 COREQ checklist.

## Abbreviations

COREQ: Consolidated Criteria for Reporting Qualitative Research
DCM: dilated cardiomyopathy
WHO: World Health Organization
