# Supplementary material for: Illness Experiences in Young and Middle-Aged Patients With Dilated Cardiomyopathy on Chinese Social Media: Qualitative Study
Source: J Med Internet Res. 2026 May 5;28:e76918. doi: 10.2196/76918 (PMC13143158; doi:10.2196/76918)
Supplement: Multimedia Appendix 1 [file jmir-v28-e76918-s001.pdf]

Contents

Supplementary Materials 1.1:Extracts from WC’s reflective journal........2

Supplementary Materials 1.2: Code List.. 7

Supplementary Materials 1.3:Additional methodology.......................... 10

These supplementary materials provide a comprehensive and transparent account of the analytical process. Supplementary Material 1.1 presents reflexive journal entries that document the researcher's ongoing thoughts and decision-making at each phase. Supplementary Material 1.2 lists the final 52 codes that emerged from this iterative process. Supplementary Material 1.3 offers a meta-perspective on the collaborative crystallization meetings that shaped the development of codes and the final themes.

Supplementary Materials 1.1:Extracts from WC's reflective journal

30.03.2025: Data Familiarization

During the data cleaning phase, I initiated the first step of analysis—familiarizing myself with the data. This involved reviewing the entire dataset and documenting my initial impressions and analytical ideas.

While reading the patients' discussion texts, I encountered strong emotional contradictions. On one hand, their descriptions of disease symptoms (e.g., "feeling suffocated," "loss of physical strength") were filled with suffering, evoking deep empathy. On the other hand, their expressions of resilience and hope, conveyed through online peer support, were equally moving. For instance, a 26-year-old patient wrote, "Every breath feels like drowning, but seeing the experiences of others makes me feel I can hang on a little longer." This tension highlighted that the narratives of young and middle-aged dilated cardiomyopathy(DCM) patients reflect not only physical and psychological struggles but also a dynamic balance between despair and hope.

Notably, many patients discussed the conflict between "family responsibility" and "self-worth" (e.g., "I don't want to burden my parents, but I fear losing them"). This closely relates to traditional Chinese values of "filial piety" and family collectivism. I began to consider how cultural values shape patients' interpretations of illness and whether this perspective might be overlooked in Western research.

02.04.2025: Crystallization Meeting – Initial Coding

Today, I held the first crystallization meeting with team members (head nurse of cardiology JJ, cardiology nursing educator (CF), graduate nursing students JL and HX, and clinician YP) to discuss a 20% random sample of independently coded data. Our goal was to examine similarities and differences in our interpretations to ensure a thorough and nuanced analysis.

I shared that my key takeaway from familiarizing myself with the data was the pervasive emotional contradiction. Others expressed similar reactions, and we all had strong emotional responses to the data. I highlighted a key observation: patients consistently described a "loss of bodily control" (e.g., "climbing stairs feels like running a marathon," "forced to sleep sitting up"), but there was significant variation in psychological coping strategies. Some patients adopted a fatalistic view, saying "Life and death are fated," while others showed intense resistance, stating, "I want to live until stem cell technology matures!" The team suggested that this difference may be age-related—young patients tend to fight actively, while middle-aged patients focus more on family responsibilities.

Nurse JJ raised an important question: "Is the 'guilt of burdening loved ones' linked to the social roles patients feel they must fulfill?" Upon revisiting the data, we found that patients' narratives revealed two distinct types of responsibility anxiety: one related to the loss of caregiving roles (e.g., "My child is still young, and I lack the strength to carry him") and the other focusing on the disruption of financial support (e.g., "How can I support my family after losing my job?"). Although gender information was not explicitly provided in the data, the expression of these responsibilities underscored patients' varied understandings of their "family roles."

05.04.2025: Coding

I have now completed most of the coding, marking the second phase of the analysis. One of the main challenges I encountered was capturing culturally specific themes within fragmented patient narratives. For instance, the code "guilt of burdening loved ones" was initially divided into subcodes like "economic burden," "loss of caregiving responsibilities," and "intergenerational guilt." However, this excessive fragmentation

led to a superficial analysis. I realized that many of these subcodes shared a common cultural core—the influence of Confucian family ethics on patients' sense of responsibility. For example, one patient wrote, "My parents sold their house to pay for my treatment. As long as I live, I owe them a debt." The term "debt" here refers not only to economic debt but also to an ethical debt related to failing to fulfill the duty of "filial piety." Consequently, we consolidated 30 related subcodes into the parent node "guilt of burdening loved ones" and defined three dimensions: economic indebtedness (e.g., "treatment costs drained family savings"), collapse of caregiving roles (e.g., "unable to care for my child"), and disruption of intergenerational responsibility (e.g., "unable to support my parents in old age"). This adjustment not only reduced redundancy but also highlighted the profound cultural impact on patients' experiences of illness.

10.04.2025: Condensing Codes

I have now reduced the number of codes to 176. Tracking the growing number of codes became challenging as the dataset revealed diverse and often conflicting viewpoints. I plan to consolidate and streamline some of the codes. For example, discussions on "integrating Chinese and Western medicine" were initially divided into 11 subcodes (e.g., "trying Chinese medicine," "regular visits to Western doctors"), which led to an analysis that focused on technical details and obscured deeper cultural insights. In the second crystallization meeting, we recognized that these codes all pointed to a central issue: how patients construct trust in treatment amid the tension between traditional and modern medicine. For instance, one patient wrote, "Western medicine controls my indicators, Chinese medicine restores my energy—both are necessary, just like the balance of yin and yang." The metaphor of "yin-yang balance" revealed the patient's cultural framework for understanding treatment, rather than a mere comparison of therapeutic effectiveness. Thus, we consolidated the subcodes into the parent node "symbiotic logic of treatment" and defined three dimensions: functional complementarity (e.g., "Western medicine is for emergencies, Chinese medicine strengthens the foundation"), cultural belonging (e.g., "drinking Chinese medicine reminds me of old remedies"), and agency in control (e.g., "trying to regain

control over my body"). This adjustment not only reduced redundancy but also emphasized patients' agency as cultural actors—they actively integrate diverse medical systems to regain control over their illness. I also had to discard ideas that, while interesting, were tangential to the research question, ensuring the codes remained focused on the core concepts.

15.04.2025: Generating Initial Themes

I have now reduced the codes to 52 and imported them into Nvivo for theme analysis. During the generation of initial themes, the relationship between "the dual collapse of family roles" and "the reconstruction of life meaning" prompted significant reflection. A representative comment from a patient was, "My life doesn't belong to me; it belongs to my parents and children—while they are alive, I cannot die." This made me reflect on the interaction between Confucian "family-centered" thinking and psychological adaptation to chronic illness. In Western research, life meaning often refers to individualistic goals (e.g., self-actualization), whereas Chinese patients place greater emphasis on "living for others." In analyzing the theme of "attitudes toward death," I realized that I focused more on positive cases (e.g., "making peace with death") and downplayed avoidance behaviors (e.g., "refusing follow-up check-ups"). This may reflect my clinical background, where I tend to favor "rational" coping strategies. I recognized the need to include the diversity of patients' coping mechanisms. For example, one 35-year-old male wrote, "I'm fine now, I don't want to go for another check-up. The more I check, the worse the results." As a result, I revised the thematic framework to include "avoidance behaviors" under "attitudes toward death" to account for these conflicting experiences.

20.04.2025: Crystallization Meeting – Developing and Reviewing Themes

In the final crystallization meeting, we discussed two themes in depth. This discussion helped me consider broader perspectives in the data.

First, regarding the theme "Multiple Challenges in the Healthcare-Seeking Process,"I propose whether it could be changed to "Unmet Support Needs." The original "Multiple Challenges in the Healthcare-Seeking Process" seems more like a discussion of issues within the healthcare system rather than the patient's personal

illness experience. Its sub-themes 1 (Misinterpretation and Delay of Early Symptoms) and 2 (Difficulty Accessing Medical Information) could be merged and categorized as the patient's insufficient understanding of disease knowledge. Head nurse JJ from the cardiology department noted that she has also observed this issue: patients' neglect and misinterpretation of early symptoms stem from a lack of relevant disease knowledge, reflecting insufficient self-management capacity regarding their health. The situation where patients are forced to transfer hospitals highlights their lack of professional guidance for continuity of care, which should therefore be distinguished from disease knowledge guidance and established as a separate sub-theme.

In the second half of the meeting, we discussed how patients reconstruct their lives. Online forums serve both as an information tool (e.g., patients searching for treatment progress) and as an emotional refuge (e.g., anonymously expressing fears). Dr. YP pointed out, "Peer support may replace traditional doctor-patient trust." This inspired me to propose in the discussion section the construction of a "clinical-digital" collaborative intervention model, such as inviting recovered patients to participate in creating health education videos that combine medical guidelines with peer narratives, conveying authoritative knowledge while maintaining emotional resonance. Ultimately, we all agreed that the theme "life reconstruction" should emphasize patients' agency—they are not passive recipients of illness but actively reconstruct their life order through "small resistances" (e.g., adjusting diet, attempting integrated Chinese and Western medicine) within the medical framework.

Supplementary Materials 1.2: Code List

In the initial coding of the dataset, the first author developed 176 codes. These codes were subsequently compressed and merged into 52 final codes prior to theme generation. The final list of 52 codes is presented below. The numbers in parentheses indicate the frequency of each code, which corresponds to the number of questions/responses assigned to that code. However, in reflexive thematic analysis, frequency is not equated with importance. Therefore, the themes were primarily generated based on their relevance to the research question.

- Difficulty breathing when lying flat, forced to adopt a seated sleeping position (9)
- Need to sit up at night to breathe, severely affecting sleep quality (3)
- The core experience of breathlessness induces a near-death sensation (1)
- Inability to complete a walk (2)
- Difficulty undressing, requiring multiple attempts (1)
- Extreme fatigue when climbing stairs (3)
- Cardiac arrest requiring emergency resuscitation (2)
- Continuous oxygen supplementation required to sustain life (1)
- Frequent blood tests to monitor disease progression (4)
- Generalized edema, significant weight gain (4)
- Night sweats, clothes soaked upon waking (2)
- Progressive decline in ejection fraction (10)
- Frequent chest tightness and palpitations (19)
- Pink frothy sputum (20)
- Wheezing sounds similar to asthma when lying flat (14)
- Decreased appetite (1)
- Fear induced by doctors' prognosis of life expectancy (94)
- Perception of life as hopeless, sinking into despair (4)
- Gradual acceptance of the incurability of the disease (16)

- Hope for medical advancements (3)
- Evading the disease by stopping medication or refusing follow-up tests (5)
- Doubts about the diagnosis, seeking to overturn it (3)
- Guilt over burdening family with caregiving responsibilities (8)
- Ending romantic relationships to reduce family burden (1)
- Abandoning plans for children due to genetic risks of the disease (2)
- Anxiety due to high medical expenses (10)
- Concern about the impact of the disease on future life (8)
- Inability to participate in social competition, feeling powerless (3)
- Social isolation, feeling lonely and helpless (3)
- Emotional fluctuations between fear, despair, and hope (4)
- Taking a leave of absence from school due to illness (12)
- Forced resignation from work (17)
- Inability to continue a sports career due to the disease (5)
- Decreased employment opportunities due to the disease (40)
- Employment limitations during job health examinations (2)
- Inability to assume financial responsibility for the family (13)
- Inability to fulfill parental caregiving responsibilities (11)
- Inability to fulfill child-rearing responsibilities (10)
- Ignoring early symptoms, delaying medical consultation (34)
- Referral to higher-level hospitals due to lack of specialists in primary care facilities (19)
- Being issued a terminal prognosis due to the severity of the condition (7)
- Experiencing financial pressure (47)
- Confusion due to lack of systematic knowledge about the disease (53)
- Desire for information on disease progression and treatment options (49)
- Desire for professional medical support (70)
- Smoking and drinking cessation to control disease progression (25)
- Maintaining a regular daily routine (33)
- Adjusting dietary habits by reducing salt and fat intake (29)

- Strict adherence to prescribed medications to control the disease (41)
- Improving physical condition through moderate exercise (17)
- Attempting integrated Chinese and Western medicine to improve health (32)
- Sharing experiences and providing mutual support through social media (46)

Supplementary Materials 1.3:Additional methodology

Details regarding crystallisation process

The analysis was led by Author 1, with both authors collaboratively engaging in the crystallization process to deepen Author 1's understanding of the data and the broader research field, thereby enhancing the credibility of the findings. Specifically, the authors met regularly throughout the analytical process to discuss similarities and differences in their interpretations of the data. This approach aimed to broaden Author 1's insights into the multiple possibilities inherent in the data and to foster awareness of her positionality. It is noteworthy that crystallization is conceptually distinct from triangulation and aligns more closely with the authors' interpretivist and reflexive stance. While both practices involve integrating multiple perspectives into the analysis, triangulation seeks to converge on a single, valid truth, whereas crystallization appreciates the complexity within the data and its multiple possible meanings. The following section details how crystallization was applied throughout the analysis.

Following the second crystallization meeting, during which the authors discussed the initial 176 codes, Author 1 undertook several steps to refine the codes into a more manageable number for the subsequent phase of analysis. Through these steps, the initial 176 codes were consolidated into a final list of 52 codes (see Supplementary Materials 2). Based on these 52 codes, Author 1 commenced the third phase of analysis: theme generation. Five preliminary themes were initially generated: (1) Bodily Control Loss; (2) Enmeshed in Emotional Turmoil; (3) Social and Family Role Disruption; (4) Multiple Challenges in Healthcare Journey; and (5) Life Reconstruction.

During the third crystallization meeting, the authors reviewed the themes and proposed revisions to the theme names. Throughout this process, in-depth discussions were held regarding the articulation of each theme. Firstly, it was observed that the

theme “Multiple Challenges in Healthcare Journey” tended to describe structural or procedural issues inherent in the healthcare system itself, rather than fully capturing the subjective experiences of middle-aged and young adults with dilated cardiomyopathy during their healthcare journeys. In other words, the original phrasing emphasized “what problems exist in the system,” failing to clearly convey the core perspective of “what patients experience and need within this system.”

Through discussions of multiple interpretations of the data, the authors recognized that the healthcare challenges faced by patients fundamentally reflect their unmet supportive needs. These needs encompass requirements for disease-related knowledge, accessibility of medical information, empathetic communication in patient-provider interactions, and expectations for continuity of care. Consequently, the theme was renamed “Support Needs in Healthcare” to more accurately capture the focal point of patients’ experiences in their healthcare journeys: they are not merely coping with challenges, but actively seeking support that genuinely addresses their circumstances.

For example, the final theme “Support Needs in Healthcare” was constructed from codes including: “Confusion due to lack of systematic knowledge about the disease (53)”, “Desire for information on disease progression and treatment options (49)”, “Desire for professional medical support (65)”, and “Referral to higher-level hospitals due to lack of specialists in primary care facilities (19)”. This demonstrates the shift from simply noting challenges to conceptualizing patients' underlying support needs.

Simultaneously, careful consideration was given to the other themes. For instance, the theme “Bodily Control Loss” was retained for its accurate reflection of patients’ core experiences with loss of physical function. The theme “Enmeshed in Emotional Turmoil” was further refined to highlight the interwoven relationship between emotional distress and physical symptoms. “Social and Family Role Disruption” emphasizes the impact of illness on patients’ social identities and family roles, while “Life Reconstruction” was adopted to encompass the dynamic process through which patients reconstruct meaning in life following their illness. Through this crystallization discussion, the articulation of themes became more closely aligned

with the patient perspectives emerging from the data, establishing a clearer conceptual framework for subsequent analysis.
